# Biomechanical effects of saddle height changes in leisure cycling with unilateral transtibial prostheses: A simulated study

**DOI:** 10.1371/journal.pone.0317121

**Published:** 2025-01-07

**Authors:** Heloisa Seratiuk Flores, Yeoh Wen Liang, Ping Yeah Loh, Kosuke Morinaga, Satoshi Muraki

**Affiliations:** 1 Graduate School of Design, Human Life Design and Science, Kyushu University, Fukuoka, Japan; 2 Faculty of Science and Engineering, Saga University, Saga, Japan; 3 Faculty of Design, Kyushu University, Fukuoka, Japan; 4 Faculty of Rehabilitation, Hiroshima International University, Higashihiroshima, Japan; Polytechnic University of Marche: Universita Politecnica delle Marche, ITALY

## Abstract

Cycling is a beneficial physical activity for rehabilitating individuals with lower-limb amputations and serves as a feasible leisure sport. However, the optimal bicycle configuration for cycling with a unilateral transtibial prosthesis at leisure levels has not been investigated. For saddle height at professional cycling levels, existing literature suggests utilizing the same configuration as that used by intact cyclists, where the knee reaches 25–35° at maximum extension. However, leisure cyclists tend to select lower saddle heights, and cycling with a unilateral transtibial prosthesis infers altered biomechanics during cycling practice. This study aimed to investigate the effects of cycling at different saddle heights with a simulated unilateral prosthesis. Ten able-bodied participants wore orthoses to simulate prosthetic conditions. The experimental task was performed on an ergometer at 40 W resistance, 60 rpm to simulate leisure cycling. Standard saddle height was defined as maximum knee extension of 45°. This height was used as the control condition and its trials were performed without orthoses. The variable heights were set as height percentage variations (-7%, -3.5%, 0, +3.5%, and +7%). Muscle activity, joint movement, force application to the pedals, perceived exertion, and comfort were evaluated. The -3.5% and -7% saddle heights resulted in joint movement and muscle activity levels closer to those in the control conditions, which also showed improved power symmetry between the affected and non-affected legs. In addition, the -3.5% height increased comfort level in participants. In conclusion, selecting lower saddle heights may be beneficial for unilateral transtibial amputees during leisure cycling. The optimal saddle height for this population may maintain maximum knee extension within the 37–45° range, dynamically measured on the affected side.

## Introduction

Loss of a lower limb significantly affects an individual’s life, introducing both physical and psychological challenges that must be addressed during rehabilitation [1, 2]. Cycling offers benefits encompassing both these challenges [3]. This activity involves lower-limb movements that are independent of one’s own weight, making it simpler to perform and suitable as a rehabilitation tool [4, 5]. Consequently, cycling can be easier than walking for lower-limb amputees, aiding in maintaining the physical conditioning of the affected limbs. Stationary cycling can also serve as initial practice for regaining walking ability using prostheses [4]. Furthermore, cycling addresses core health issues such as vascular disease and diabetes, which are the leading causes of amputation in well-developed countries [6, 7]. It also helps combat sedentary lifestyles, which disproportionately affect lower-limb amputees [8]. Additionally, cycling promotes psychological well-being by demonstrating patients that they can regain lower-limb functionality, which may be daunting when first using a prosthesis for walking.

Despite these benefits, the equipment requirements for persons with lower limb amputations during cycling are not fully understood. One of the most common types of lower-limb amputation affects a single side at a below-the-knee level: transtibial unilateral amputation [9]. Owing to the differences in movement between the intact and affected limbs, cycling with a unilateral transtibial prosthesis can lead to discomfort [3] that can be mitigated by specific bicycle settings or prosthetic devices. Recent studies have examined prosthesis design and foot placement on the pedal [10]. For bicycle configurations, the guidelines for non-amputees may be applicable to lower-limb amputees without comorbidities [4]. However, cycling movement is altered in individuals with a prosthesis primarily because of the absence of ankle joint movement, which is typically rigid in walking prostheses and absent in professional-level cycling prostheses. This difference can cause asymmetries, making cycling uncomfortable [4]. When using a unilateral prosthesis, the leg with the prosthesis must extend further to the bottom pedal position to compensate for the lack of ankle movement. Meanwhile, in the highest pedal position, the knee and hip joints may flex more compared with an intact leg because of the absence of ankle dorsiflexion [11]. These exaggerated joint movements lead to asymmetry between the intact and prosthetic limbs, potentially causing other musculoskeletal disorders [12, 13].

Previous studies have explored mitigating this asymmetry by altering the crank length on the affected side [14], which reportedly increases the comfort of prosthesis-wearing cyclists unfamiliar with cycling [4]. However, bicycle cranks are only commercially available at specific lengths, which are not highly customizable and may not fit individual anthropometric dimensions. One way to emulate crank shortening without special parts is to alter the saddle height, allowing for more precise adjustments. In settings such as elite cycling, athletes often wear cycling-specific prostheses, and use a higher saddle height, compatible with able-bodied elite cycling, while possibly utilizing the shorter crank on the affected side [14]. This higher saddle height enables more effective use of muscle lengths and joint power [15, 16]; therefore, the lower limbs reach an ideal setting for power production, at the cost of kinematic symmetry, inferred by the unilateral shorter crank and rigid cycling prostheses. In contrast, leisure cycling often employs suboptimal heights. Leisure cycling involves shorter activity periods, lower resistance, and slower cadences, thus minimizing the risk of acute and overuse injuries [17]. Additionally, leisure cyclists tend to prefer lower saddle heights, which can improve maneuverability and stability [18].

In the context of unilateral prostheses, the lower saddle heights can minimize the use of the ankle joint on the affected side; thus, making its kinematics more similar to that at the prosthetic side, which often has a rigid ankle joint. This would come at the cost of reduced power production ability; however, leisure cycling does not reach the same levels of power requirements as elite cycling. Potentially, the lower saddle height could lead to increased comfort stemming from the improved movement symmetry. Therefore, it can be theorized that leisure cycling with unilateral transtibial prostheses would benefit from either the higher saddle height employed in elite cycling, or lower saddle height already commonly used in leisure cycling.

This study aimed to evaluate the biomechanical effects of leisure cycling at different saddle heights using a simulated unilateral transtibial prosthesis. To achieve this, biomechanical data were collected between affected and unaffected legs, pertaining kinetic and kinematic aspects. Parameters involved the muscle activity of the gastrocnemius medialis, vastus medialis, and semitendinosus, joint movement of hip and knee, symmetry of force applied to the pedals, as well as torque effectiveness and pedal smoothness, and subjective feedback on perceived exertion and comfort. To elucidate the effects of the different saddle heights, results were compared between simulated prosthetic cycling conditions and a control, intact cycling condition.

## Methods

### Participants

Ten female (n = 6; age: 27.5 ± 2.7 years; height: 157.6 ± 1.9 cm; weight: 59.0 ± 10.8 kg) and male (n = 4; age: 28.3 ± 3.6 years; height: 167.7 ± 2.7 cm; weight: 67.0 ± 8.4 kg) able-bodied participants were recruited between August 1^st^, 2023, and August 20^th^, 2023. The inclusion criteria consisted of being between the ages of 25 and 35 years and practicing leisure or commuting cycling with at least monthly regularity. Using cycling as physical training or sports practice and presenting any condition that could affect the practice of cycling or could worsen during the experiment were exclusion criteria. In addition, the participant height was limited to 172 cm because of experimental setting limitations on saddle height variance.

Participants answered a health and bicycle-riding habit questionnaire that enforced the exclusion criteria, and provided written informed consent to participate in the study. Leg dominance was assessed through asking the question “if you were to kick a ball, which leg would you use” [19], with the majority of participants reporting right-leg dominance (n = 9). Anthropometric data was then collected, including the length of the right leg inseam (75.1 ± 2.4 cm) and foot length (23.7 ± 1.1 cm).

This study was approved by the Ethics Committee of the Faculty of Design, Kyushu University (approval number 532).

### Simulated prosthesis condition

To collect data comparable to intact cycling and standardize the results, participants without amputations were recruited for the experiment. The participants wore custom orthoses (Arizono Orthopedic Supplies Co., Ltd., Kitatkyushu, Japan) simulating prosthetic conditions, which prevented the biological foot from contacting the pedal and ankle from exerting force throughout the crank cycle. The inability to contact the pedal also simulated the lack of tactile feedback, experienced by prosthesis users. This simulated prosthetic condition allows for the isolation of ankle-movement factors and minimizes possible influence of amputation and disability levels. Furthermore, it also allows a better evaluation of the adaptation to the prosthesis. The orthoses were worn on the right side and attached below the knee, designating the right leg as the affected leg (AL) and left leg as the unaffected leg (UL). The orthoses featured an aluminum strut connected directly to the pedal via a cleat, aligning the foot in a mid-foot position. Previous research suggests that this position can help mitigate the asymmetry between intact and amputated limbs [10]. After the donning of the orthosis, participants were given up to 5 min to practice cycling with it.

### Saddle height setting

Based on previous cycling research, the ideal saddle height is achieved when the knee is at 25–35° extension with the crank at the 180° position (bottom of the cycle) [15, 20]. This setting optimizes power output and muscle performance and is suitable for physical training or competitive cycling. However, for leisure cycling, where the power output is lower, cyclists may prefer a lower saddle height. In this experiment, the standard saddle height was set to achieve a 45° knee angle at the bottom of the cycle, measured statically with a goniometer and corresponding to 46° ± 3° when assessed dynamically.

#### Experimental conditions

**Fig 1:**
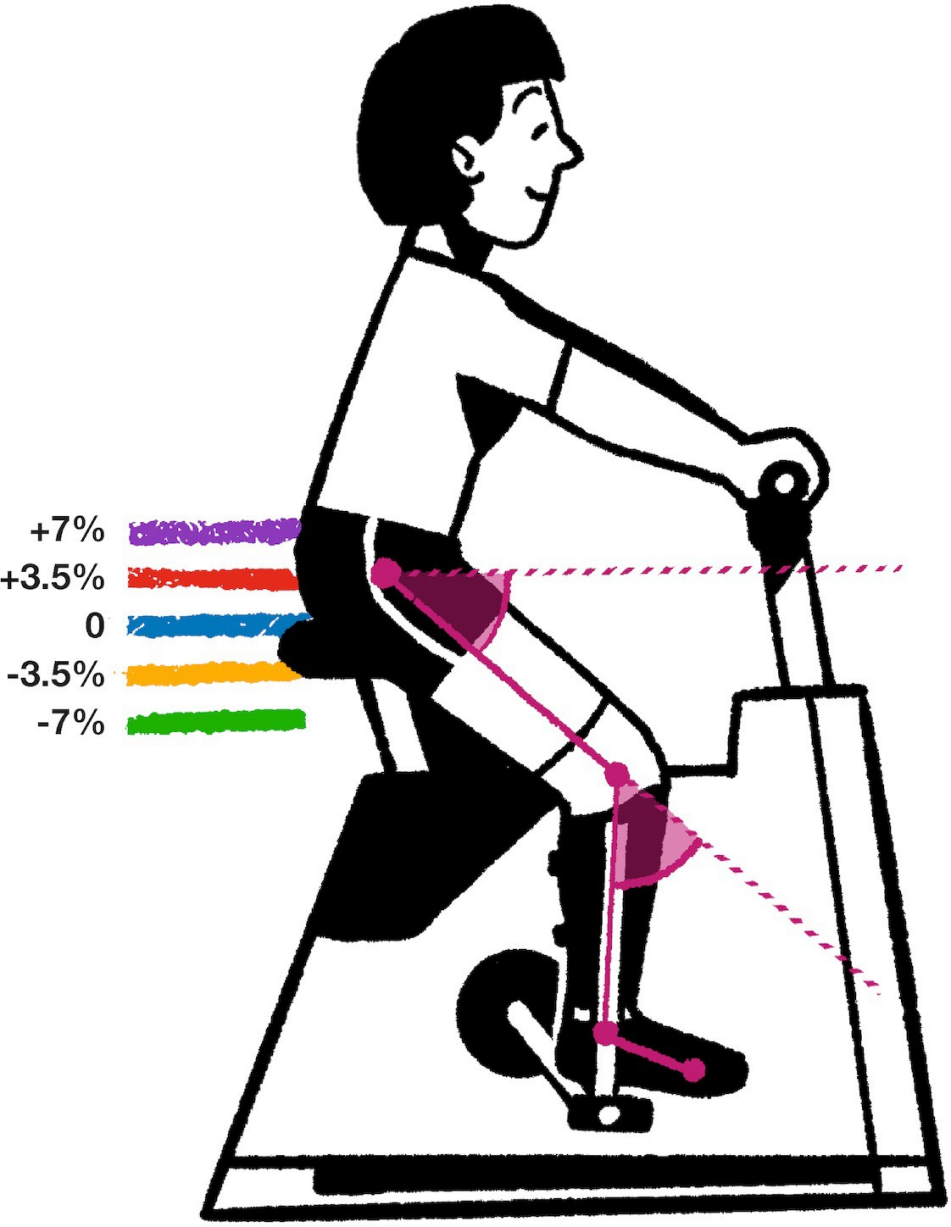
Graphical representation of the saddle heights used during the experiment (+7%, +3.5%, 0, -3.5%, and -7%), tracking points, and calculated angles. **Control:** Regular cycling without orthosis. **0:** Employing the orthosis, participants cycled at the same saddle height as in the control condition. **-7%, -3.5, +3.5, and +7%:** Saddle height variations calculated over the control/0 height, performed while wearing unilateral orthosis.

The different heights of the saddle to be evaluated were measured from the top of the saddle to the crank axis and set as variants of 3.5% (2.31 ± 0.07 cm) of the standard height, based on previous research [21]. Therefore, the conditions for the experiment were defined as (Fig 1):

### Experimental setup

**Fig 2:**
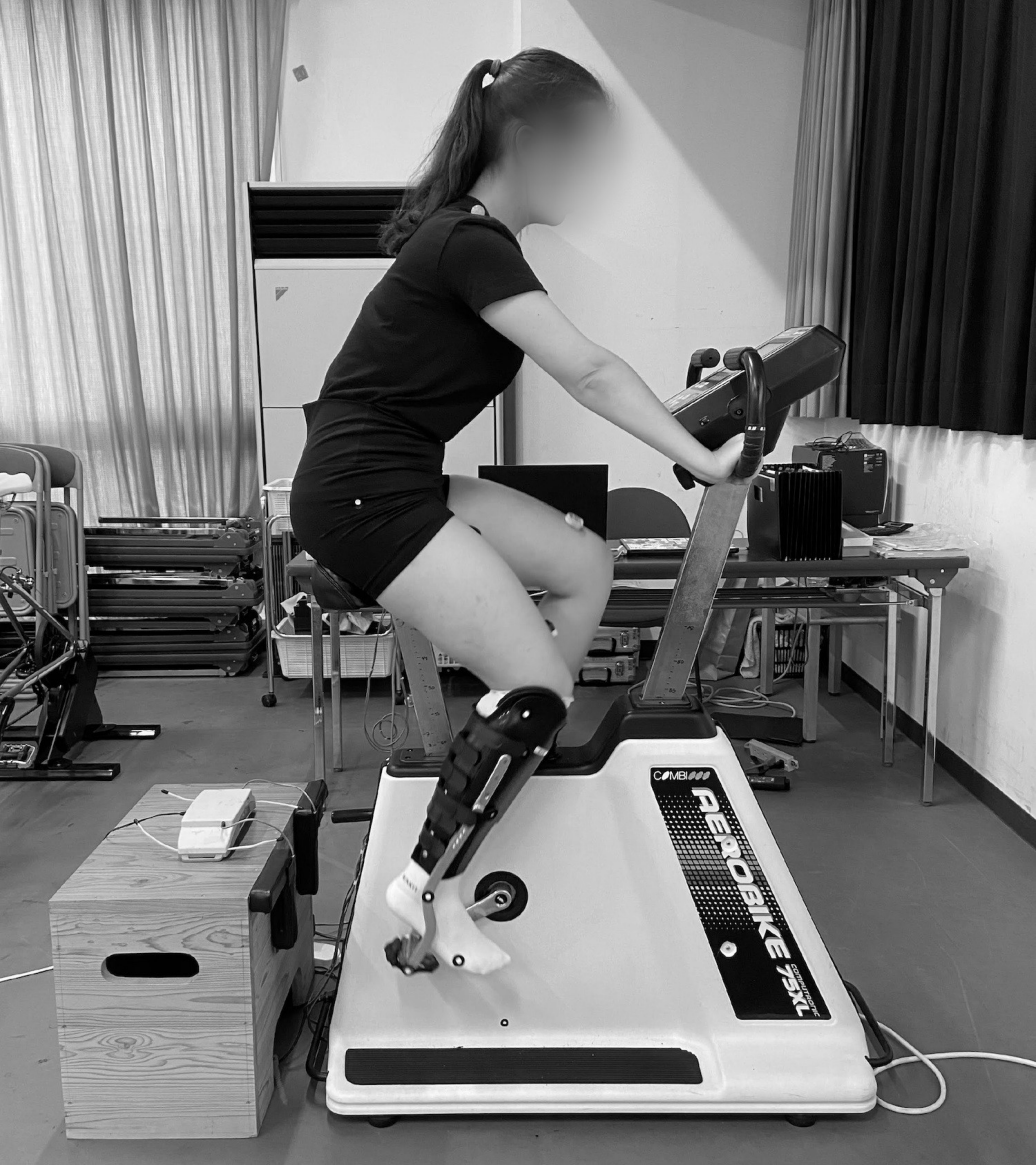
Experimental environment.

Experiments were conducted in an air-conditioned room kept at approximately 22°C. The participants cycled on an AeroBike 75XL ergometer (Konami Sports Co., Ltd., Kanagawa, Japan) with a crank length of 170 mm (Fig 2). The ergometer was modified to include Assioma Duo power-meter pedals (Favero Electronics Srl., Arcade, Italy). The handlebar height was set to 100 mm above the saddle height in all the conditions.

Clipless pedals were equipped with three-dimensional (3D)-printed attachments to function as platform pedals, complemented by thermoplastic fastening to limit the forward foot position. This limiter prevents upward pedal pulling, which is possible with clipless pedals. The 3D-printed attachments were used on both sides in the control condition and only on the unaffected side in subsequent trials. The orthosis was directly connected to the pedal via a cleat.

### Experimental protocol

For the experiment, the participants wore tight-fitting clothing (t-shirt and shorts) and ankle-covering socks. Laceless MW100 trainers (New Balance Athletics Inc., Boston, MA, USA) of appropriate sizes were provided. These trainers were used during the control condition and only on the unaffected side during the orthosis conditions. Cycling was performed with 40 W resistance at 60 rpm, simulating everyday leisure cycling. A metronome ensured consistent cadence.

Each experimental trial lasted 2 min, with three replicates for each of the six conditions. The conditions were randomized except for the control condition, which was always performed first. Breaks of 30 s were enforced between trials, and of 3 min between conditions. The first trial of each condition served as practice, with data being collected during the latter two trials. The first and last 30 s of each trial were discarded, and data were analyzed for 10 consecutive crank revolutions.

### Measurements

#### Joint angles

Two-dimensional motion analysis was conducted using a Panasonic HC-300M camera (Panasonic Co., Osaka, Japan) which recorded at 30 frames per second and 1080p resolution. The camera was positioned 3.8 m from the ergometer on the participant’s affected (right) side. A secondary camera (HDR-CX560V; Sony Co., Tokyo, Japan) with identical capture settings was placed 1 m from the unaffected side for data verification. Four color-contrasting self-adhesive markers were symmetrically placed on anatomical landmarks: the greater trochanter, lateral femoral epicondyle, lateral malleolus, and fifth metatarsal head, corresponding to the hip, knee, ankle, and foot, respectively. Ankle joint movement was collected for monitoring reasons and was not used in the study. An additional marker was placed on the pedal to segment the data for each complete cycle.

Video footage was processed using Adobe Premiere Pro 15.4 (Adobe Inc., San Jose, CA, USA). Motion analysis was performed using motion analysis software [22] that employed a Discriminative Correlation Filter Tracker with Channel and Spatial Reliability [23] algorithm for automatic motion tracking. Each frame was verified manually. The angles were calculated using software, as illustrated in Fig 1.

#### Muscle activity

Muscle activity was recorded using a telemeter surface electromyography (sEMG) system (WEB7000; NIHON Kohden Co., Tokyo, Japan) at 1 kHz. The signal was internally bandpass-filtered (15–500 Hz) and rectified. Following the Surface Electromyography for the Non-Invasive Assessment of Muscles guidelines [24], four electrodes were placed bilaterally on the proximal portion of the legs to monitor the vastus medialis and semitendinosus muscles. An additional electrode was placed on the gastrocnemius medialis muscle of the UL. These muscles were specifically chosen based on previous research, with the vastus medialis, semitendinosus, and gastrocnemius showing stronger changes in activity under different unilateral prosthesis cycling conditions [10, 14, 25, 26]. A direct-current signal sampled wirelessly at 1 kHz was correlated with a Hall sensor designating the crank orientation. After collection, an 8 Hz low-pass linear envelope filter was applied to smooth the rectified sEMG signal [27], reflecting the magnitude of muscle activity. The data were normalized to the control condition and segmented into cycles using Hall sensor data.

#### Instrumented pedals

Assioma Duo instrumented pedals were installed bilaterally on the ergometer to assess the force applied to the pedals [28]. Data were recorded on a computer using an ANT+ receptor at 1 Hz using GoldenCheetah ver. 3.6 software. The parameters measured included cadence and power, which were used to monitor the participants’ adherence to target power settings and cadence. Left and right balance assessed the contribution of each leg to a full revolution of the crank, with values closer to 0 indicating a higher contribution from the right leg and those closer to 100 indicating that the left leg was more prominently used. The torque effectiveness measures the influence of the force applied to the pedals on the resulting force vectors, with values close to 100 indicating the greatest effectiveness. Pedal smoothness was calculated as the constant application of power to the pedals throughout the cycle, with constant and uniform power delivery achieved at 100. Torque effectiveness and pedal smoothness were measured independently on each side.

#### Subjective evaluation

Thirty seconds prior to the conclusion of each trial, the participants were asked to assess their perceived exertion using Borg’s 6–20 RPE scale [29], which was visibly positioned on a wall in front of the ergometer. During the practice trial for each condition, the participants were instructed to evaluate their comfort level in comparison to that during the immediately preceding condition and indicate the height at which they were most comfortable at that point. Additionally, during the control trial, participants who owned a bicycle (n = 7) were questioned whether the standard saddle height resembled that of their regular bicycle, with four participants stating that it was the same, two indicating that it was lower, and one reporting that it was higher.

### Statistical analysis

One-way repeated analysis of variance (ANOVA) was performed using SPSS Statistics (version 21.0; IBM Co., Armonk, NY, USA) to investigate the effects of different saddle heights on the mean values of all parameters for each participant. The factors were established as the five different heights (-7%, -3.5%, 0, +3.5%, and +7.5%). To ensure the validity of repeated-measures analyses, the assumption of sphericity was tested using Mauchly’s test. When violated (p < 0.05), Greenhouse–Geisser corrections were applied. Additionally, Dunnett’s test was performed to investigate the significance of differences between simulated prosthetic conditions and the control condition. Dunnett’s test was not conducted for the muscle activity results because the control condition was used for normalization. To evaluate the joint angles throughout the cycling movement, statistical analysis was conducted between the means at the following crank positions: 0, 90, 180, and 270°. Pairwise comparisons were performed using Bonferroni adjustment. Statistical significance was set by α = 0.05, with descriptive statistics being used for 95% confidence interval (CI). Results for pedal, muscle activity, and perceived exertion are represented as an average ± standard deviation. The joint angles and muscle activity relative to crank angle results are displayed as averages.

## Results

### Joint angles

#### Knee

**Fig 3:**
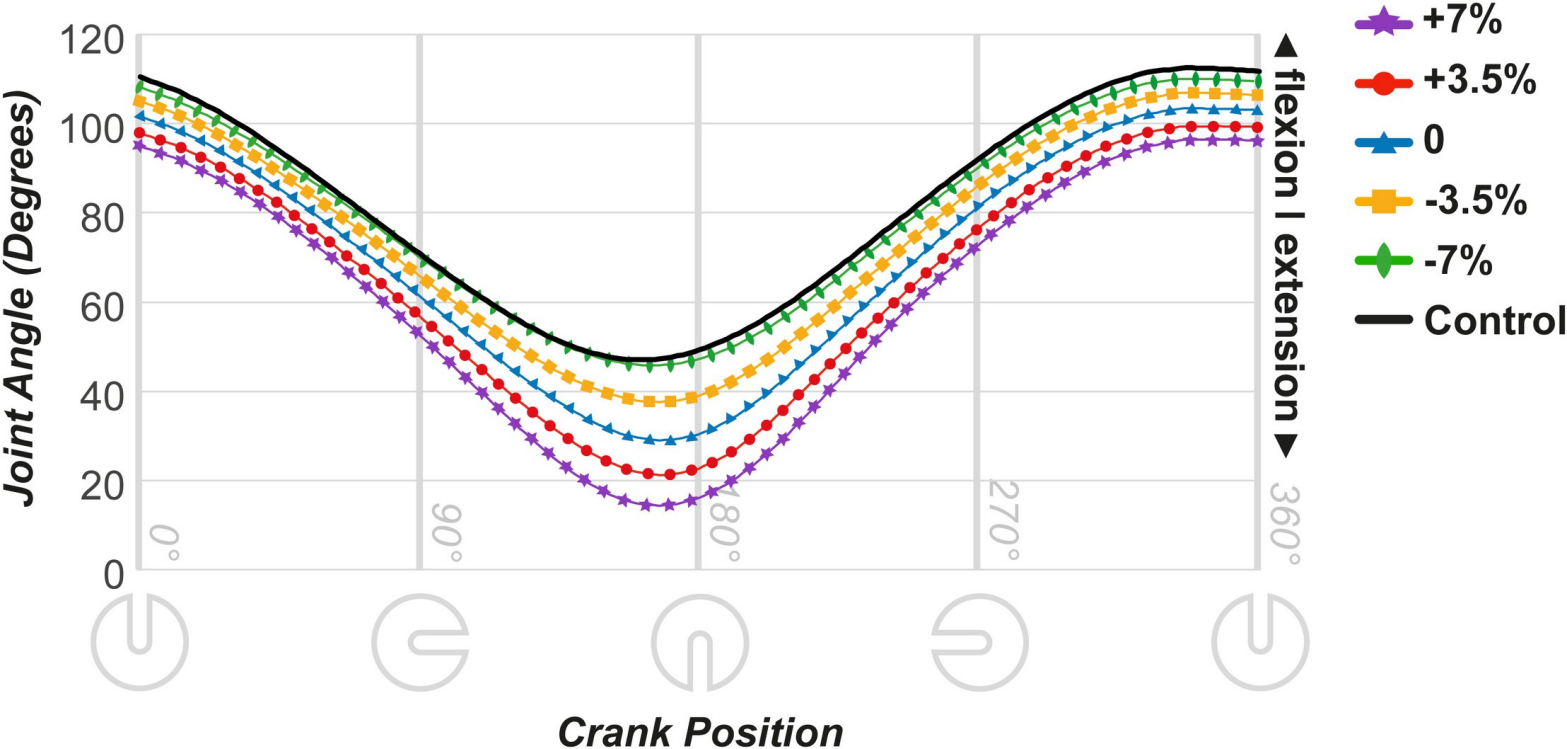
Joint movement data throughout the cycle in the affected leg for the knee joint.

The knee joint angles are shown in Fig 3. The -7% saddle position exhibited joint movement closest to the control. ANOVA revealed a significant effect of saddle height on the knee joint angles throughout the cycle (0° = F [4,36] = 231.92, p < 0.001; 90° = F [4,36] = 233.51, p < 0.001; 180° = F [1.88,16.90] = 314.88, p < 0.001; 270° = F [4,36] = 408.86, p < 0.001). Pairwise comparisons indicated significant differences between all saddle heights throughout the crank rotation (p < 0.05). The +7% height differed the most from the control and exhibited the highest extension at the 180° crank position, and the -7% height showed the most flexion throughout, comparable to the intact cycling levels. Dunnett’s test revealed significant differences between the control condition and the -3.5%, 0, +3.5%, and +7% conditions throughout the cycle, all of which showed more extension than that of the control, with only the -7% condition showing no significant difference from the intact cycling levels (S1 Appendix).

#### Hip

**Fig 4:**
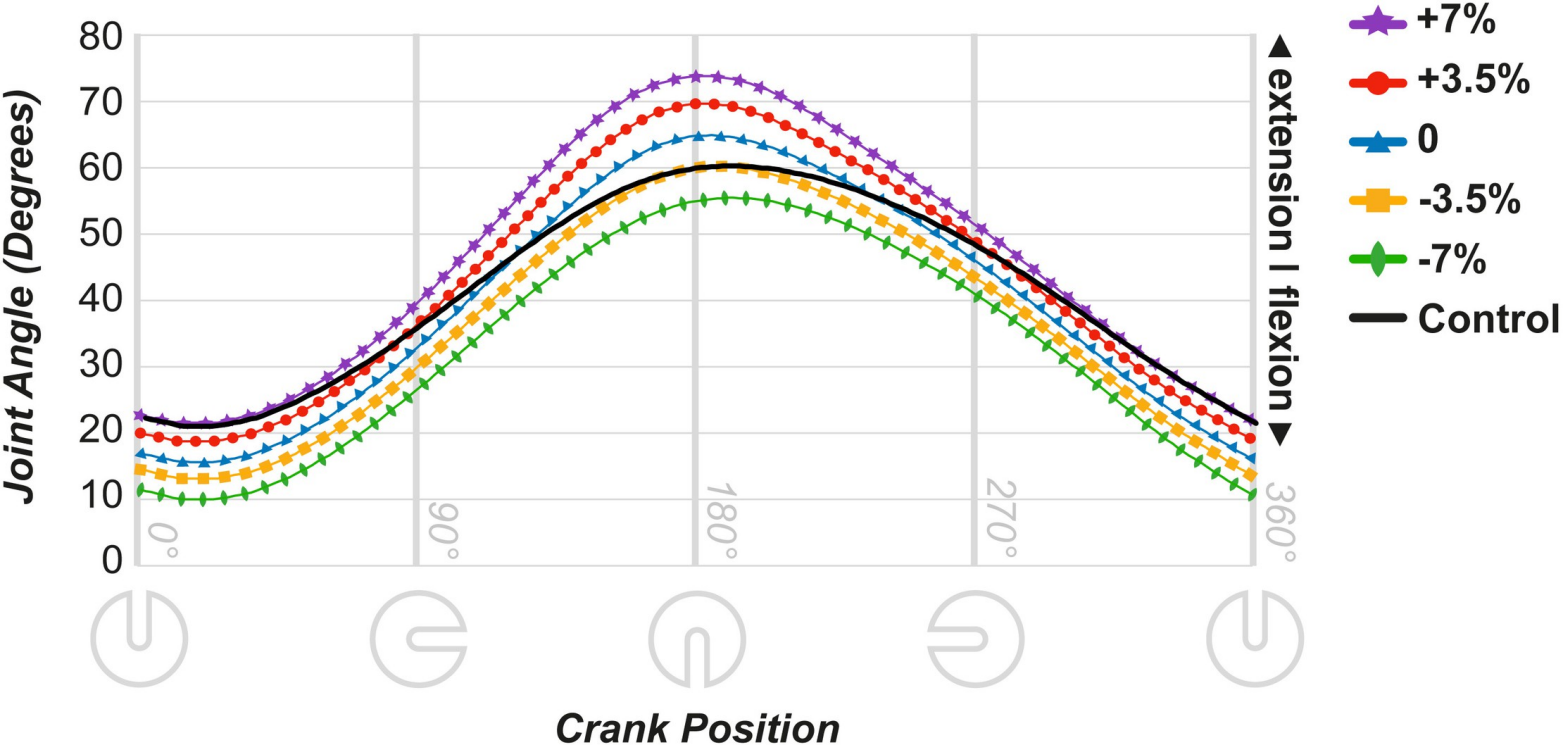
Joint movement data throughout the cycle in the affected leg for the hip joint.

Fig 4 shows the hip joint movement. In the 0° crank position, hip flexion was closer to the control values at +7% saddle height. When the crank was at the bottom dead center position (180°), -3.5% height movement became the closest to control. Similar to the knee joint mean angles, the ANOVA results indicated a main effect of saddle height across all measured crank angles (0° = F [4,36] = 337.25, p < 0.001; 90° = F [4,36] = 410.98, p < 0.001; 180° = F [1.46,13.18] = 302.17, p < 0.001; 270° = F [4,36] = 259.95, p < 0.001). Post-hoc analyses revealed that for the whole cycle, differences between the saddle height means were significant (p < 0.01), with the +7% height exhibiting the most extension throughout, peaking at the 180° crank position, and the -7% height showing the most flexion. Dunnett’s test results (S1 Appendix) revealed that at the start of the cycle, saddle heights of -7%, -3.5%, 0, and +3.5% significantly differed from the control, with +7% showing flexion levels similar to those of intact cycling. At 180° (lowest pedal position), -7%, 0, +3.5%, and +7% heights differed significantly, whereas -3.5% closely matched control extension levels. At 90° and 270°, which are flexion-extension transition points, all heights significantly deviated from the control. Overall, all saddle heights exhibited significant differences at specific cycle points, with the +7% height being closest to the control in flexion at the start and end, and -3.5% height aligning at mid-cycle during peak extension.

### Muscle activity

**Fig 5:**
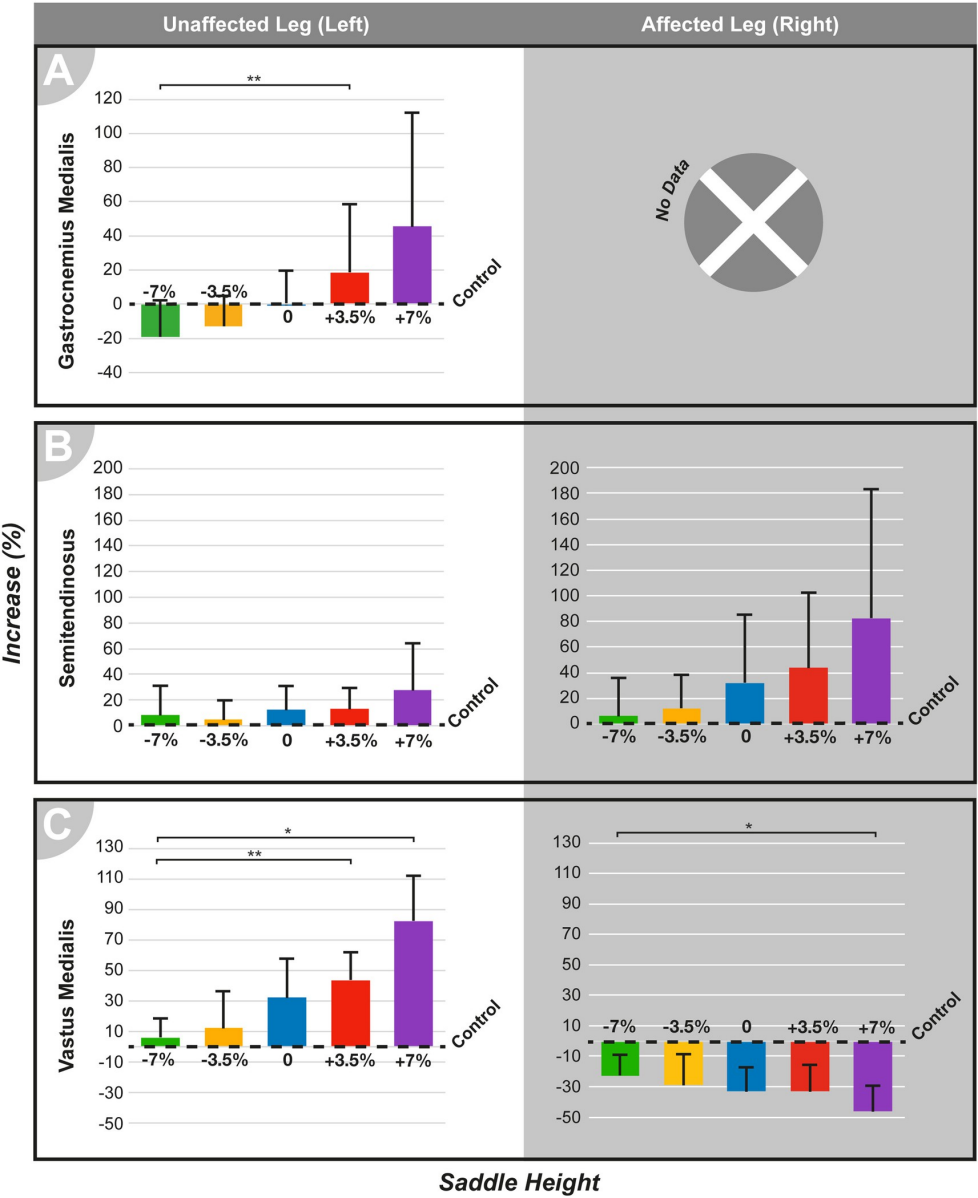
Mean muscle activity for all seat heights in both the unaffected and affected legs. Control condition is set as baseline (0) and is indicated. (A) Gastrocnemius medialis; (B) Semitendinosus; (C) Vastus medialis. **p < .01, *p < .05, compared between the height conditions.

**Fig 6:**
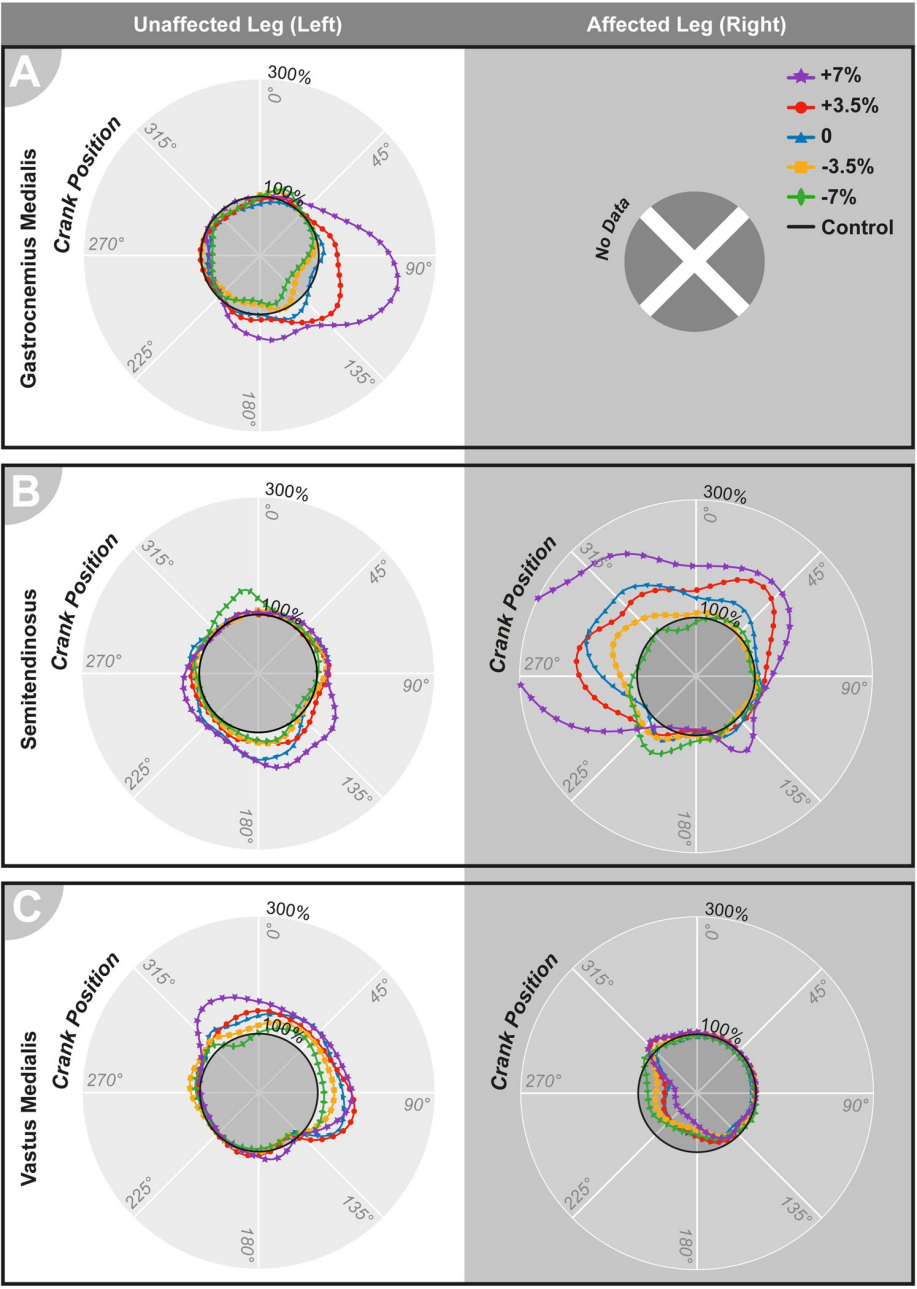
Mean muscle activity in the unaffected and affected legs in relation to the crank angle. (A) Gastrocnemius medialis; (B) Semitendinosus; (C) Vastus medialis.

EMG data were calculated as % variation over the control condition and are shown as means in Fig 5. These data are plotted in a circular graph, as shown in Fig 6. The dotted line in the center of the graph represents the mean muscle activity in the control condition. Lines correlating with the conditions represent a percentage increase or decrease over the control. The degrees of circumference in the graph correspond to the crank position, and muscle activity refers to the represented positions. ANOVA was conducted with the overall mean muscle activity calculated over each cycle.

#### Gastrocnemius medialis

Mean results for the gastrocnemius medialis (Fig 6A) in the UL indicated that altering the saddle height from 180° to 360° had minimal impact on muscle activity. However, between 90° and 135°, a significant increase in muscle activity was observed for higher saddle heights of +3.5% and +7%, with average increases of 19% and 46%, respectively, throughout the cycle. Visual inspection showed that the 0% and -3.5% heights were closer to those of the control. ANOVA for the overall means (Fig 5A) indicated a significant effect of saddle height (F [4,36] = 7.26, p < 0.001). The +7% height showed the greatest overall increase, with the +3.5% height significantly differing from -7% (p < 0.01, 95% CI = 0.08, 0.38).

#### Semitendinosus

In the UL, muscle activity levels for the semitendinosus (Fig 6B) showed no significant alteration under different saddle heights, as corroborated by ANOVA (Fig 5B): no main effect of saddle height was found. The results were different in the AL, with most saddle heights leading to increased muscle activity (6–83%), especially from 225° to 90°. The +7% saddle height showed the greatest increase, with +3.5% and 0% presenting similar levels of muscle activity above the control condition. Heights -7% and -3.5% showed levels closer to the control. Similar to the UL, the ANOVA results indicated main effect of saddle height (F [2.12,19.09] = 4.36, p < 0.01), but the Bonferroni-corrected pairwise comparisons revealed no significant differences between the height conditions.

#### Vastus medialis

Muscle activity levels in the UL were increased in the vastus medialis (Fig 6C) in all saddle height conditions (9–40%), with the highest levels of increase observed in the +7% condition. The most reduced saddle height, -7%, presented results closest to control. The main effect of the saddle height (Fig 5C) was found through ANOVA (F [4,36] = 7.26, p < 0.001), and pairwise comparisons revealed a significant difference between the heights -7%, which showed levels closest to those of the control, and both +7% (p < 0.05, 95% CI = -0.62, -0.01) and +3.5% (p < 0.01, 95% CI = -0.38, -0.08), which presented the strongest increase. The AL showed an overall decrease in the mean muscle activity (-46–-23%), more notably occurring between 135 and 315°. The +7% height results deviated the most from those of the control group, showing the greatest decrease. Statistical analysis showed a significant effect of saddle height (F [4,36] = 7.87, p < 0.001), with a significant difference between the -7% height, which was the closest to the control, and the +7% height, which showed the most reduced levels of muscle activity (p < 0.05, 95% CI = 0.04, 0.44).

### Instrumented pedals

**Fig 7:**
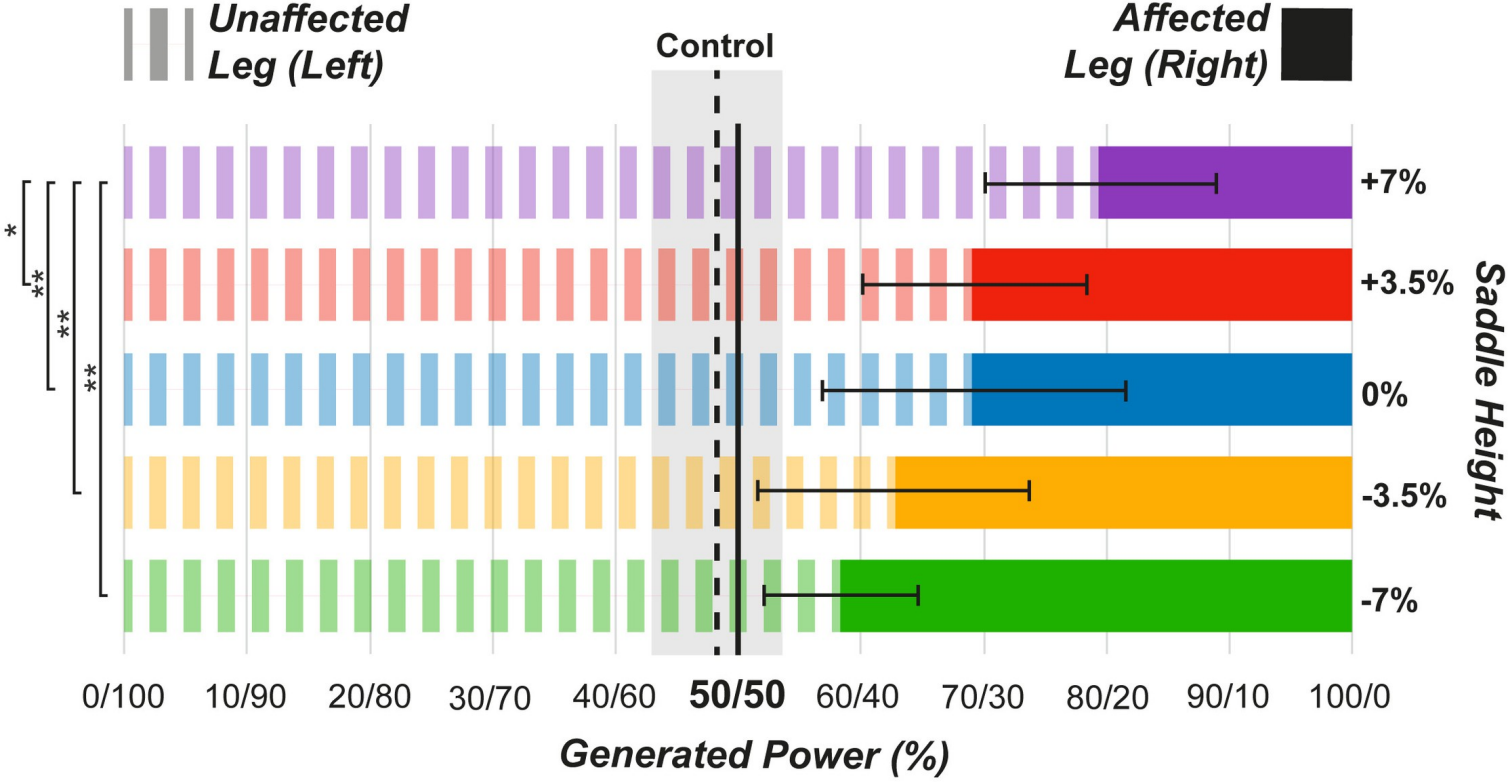
Generated power percentage for left (unaffected leg) and right (affected leg) balance. ***p < .001, **p < .01, *p < .05 compared between the height conditions.

**Fig 8:**
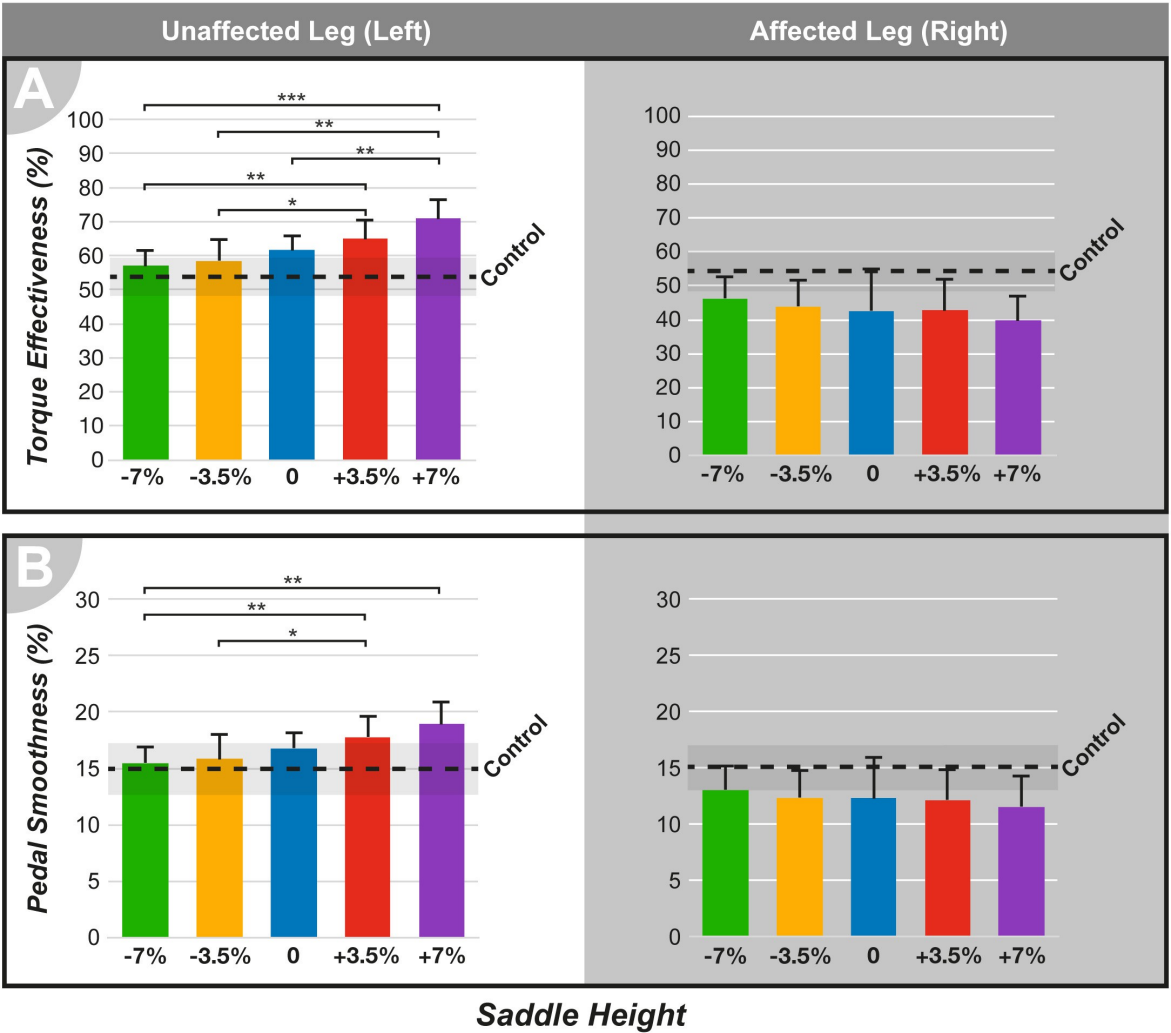
Instrumented pedal data means for the unaffected and affected legs. (A) Torque effectiveness, (B) Pedal smoothness. ***p < .001, **p < .01, *p < .05, compared between the height conditions.

Fig 7 illustrates the mean percentages of the left and right balance. Fig 8 shows the mean percentages of torque effectiveness, and pedal smoothness. For left and right balance (Fig 7), ANOVA revealed a significant effect of saddle height (F [4,36] = 16.13, p < 0.001). The +7% height exhibited the most asymmetry, significantly differing from all other saddle heights (-7% = p < 0.01, 95% CI = 10.24, 32.01; -3.5% = p < 0.01, 95% CI = 4.63, 28.50; 0 = p < 0.01, 95% CI = 3.50, 17.06, and +3.5% = p < 0.05, 95% CI = 0.39, 19.65), which were more symmetrical. However, Dunnett’s test results (S1 Appendix) showed that all heights were significantly different from the control, which exhibited the most symmetry.

In the UL, the mean torque effectiveness (Fig 8A) was increased with saddle height. Statistical analysis indicated significant differences between the groups (F [4,36] = 23.74, p < 0.001). While all heights showed increased torque effectiveness, the +7% condition deviated the most from the control, presenting the highest increase and significantly differing from -7% (p < 0.001, 95% CI = 7.20, 20.44), -3.5% (p < 0.01, 95% CI = 4.23, 20.57), and 0 (p < 0.01, 95% CI = 3.85, 14.67). This trend extended to the +3.5% condition, which also significantly differed from -7% (p < 0.01, 95% CI = 2.94, 12.70) and -3.5% (p < 0.05, 95% CI = 0.74, 12.04). The AL showed no main effect of saddle height. In the UL, Dunnett’s test (S1 Appendix) showed no significant differences between the -7% saddle height and control. In the AL, all heights showed results that were significantly different and lower than those of the control.

For pedal smoothness (Fig 8B), ANOVA indicated a main effect of saddle height on the UL side (F [4,36] = 12.78, p < 0.001). The +7% height, similar to the torque effectiveness, showed the strongest increase in pedal smoothness and was significantly different from the reduced saddle height -7% (p < 0.01, 95% CI = 1.47, 5.39), which had percentages closer to those of the control. The +3.5% height also showed significant differences between the -7% (p < 0.01, 95% CI = 0.83, 3.69) and -3.5% (p < 0.05, 95% CI = 0.08, 3.74) heights, with the -7% height showing overall lower levels of pedal smoothness. In the AL, no main effect of saddle height was found. Dunnett’s test (S1 Appendix) showed no significant differences between the -7% and -3.5% saddle heights and control in the UL. For the AL, results for all heights significantly differed from the control, showing lower levels of pedal smoothness.

### Subjective evaluation

When asked to subjectively choose the most comfortable saddle height, one participants chose the -7.5% height, and another three chose the standard 0 height. One participant chose a +3% increase in height. Most participants (n = 5) elected the -3.5% height as the most comfortable.

**Fig 9:**
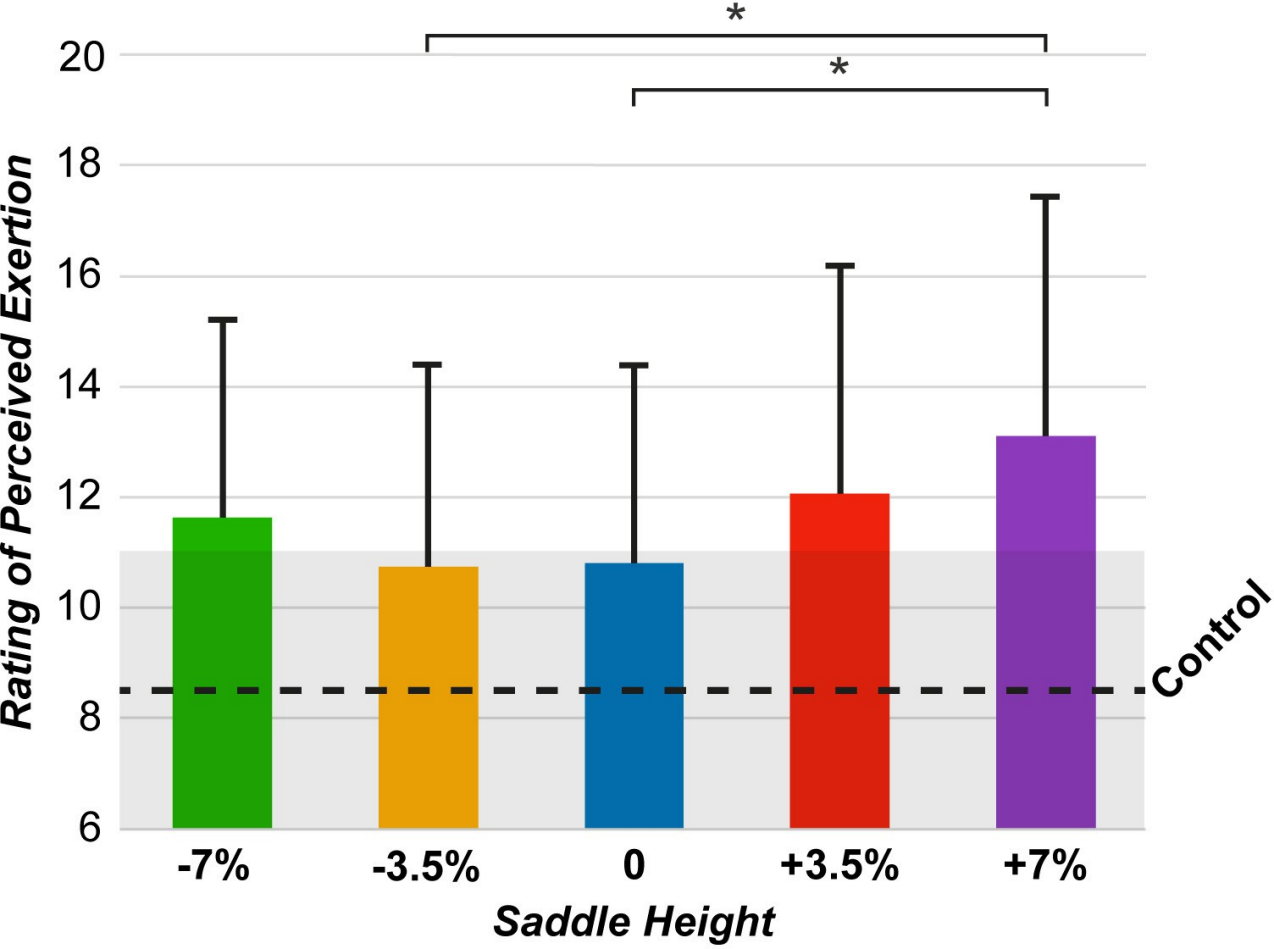
Mean rating of perceived exertion. *p < .05, compared between the height conditions.

Perceived exertion was also evaluated using a 6–20 points Borg scale, the means of which are shown in Fig 9. ANOVA showed the main effect of the saddle height (F [4,36] = 10.71, p < 0.001), with post-hoc analyses showing significant differences between the height that was perceived as the most strenuous, +7%, and the heights -3.5% (p < 0.05, 95% CI = 0.33, 4.40) and 0 (p < 0.05, 95% CI = 0.40, 4.20). Additionally, Dunnett’s test (S1 Appendix) showed significant differences between all experimental conditions and the control condition, all of which presented higher scores than control.

## Discussion

This study assessed the effect of varying saddle heights on movement and power asymmetries during cycling under simulated unilateral transtibial prosthesis conditions. This novel evaluation in the context of leisure cycling offers valuable insights into optimizing bicycle fit in individuals with unilateral amputations. A previous study with this population employed the LeMond method for saddle height adjustment [10, 30], resulting in heights similar to the +3.5% and +7% conditions used in this study. Other studies on professional cycling with amputations [14, 26, 31] either utilized methods designed for intact cyclists or allowed cyclists to set saddle heights at their own discretion. Thus, while the effects of saddle height changes in intact cycling are known, an evaluation of the impact of these configuration changes in cycling with prostheses has not been conducted thus far, either in elite or leisure cycling contexts. Furthermore, it can have great influence on the biomechanics of cycling, as well as perceived comfort.

### Effects of raising the saddle height

According to previous research on professional cycling, the recommended saddle height that provides optimal power output would result in a knee angle of 25–35° at the bottom of the cycle, or at its maximum extension [20, 21]. This saddle height is commonly applied in road cycling at both professional and amateur levels. Other methods of selecting the saddle height [17, 30] also result in near-full knee extension at the bottom of the cycle. This higher saddle heights offers numerous benefits such as reduced knee overuse injuries and improved cycling performance [32, 33].

#### Changes in pedaling technique

In the AL, the 25–35° maximum knee angle joint range was achieved at the 0% saddle height (29°) because of the lack of ankle joint movement in the simulated prosthesis condition. Increasing the saddle height led to a greater deviation of the kinematic parameters from the control condition. As shown in Fig 3, at the peak extension, the knee joint reached 14° at +7% height and 21° at +3.5% height. Similarly, a higher extension was observed in the hip angle (Fig 4). Peak extension at the 180° pedal position was higher than that in the control under the +3.5% and +7% conditions, which was corroborated by Dunnett’s test results.

The kinetic changes due to the increased saddle height may be linked to a difference in the pedaling technique, as evidenced by the sEMG results. Starting at the beginning of the cycling movement, according to the pedal position of the AL, changes in the muscle activity in both legs were as follows. During the first and second quarters, muscle activity remained similar to that of the control. In the third quarter, the vastus medialis (Figs 5C and 6C) showed increased activity on the UL side and reduced activity on AL side. Both the increase and reduction were exacerbated under the +7% and +3.5% conditions, suggesting that, with increased saddle height, the UL becomes the primary driver of knee extension, whereas AL relies on the increased activity of the UL to complete the cycling movement, thus increasing the asymmetry in muscle activity. This pattern was also noted in a previous study [10] but contrasts with findings in high-resistance unilateral amputee cycling, which described increased muscle activity in the vastus medialis of the AL [14, 31, 34].

During the fourth cycling quarter of the AL, the gastrocnemius medialis muscle (Figs 5A and 6A) became more active in the UL, peaking at a 250% increase over the control condition at +7% height. As mentioned previously, the higher saddle heights provide many advantages to professional cyclists and also enable better use of the involved muscles’ effective length [35]. Previous studies [36, 37] on intact cycling have shown that higher saddle heights increase gastrocnemius muscle activity, similar to the effects observed here. This suggests that regardless of the simulated prosthesis conditions, higher saddle heights allow the UL reach an optimal position within the maximum knee extension range of 25–35°. Consequently, gastrocnemius activity increases as plantar flexion is used more extensively at the bottom of the cycle, in contrast to that at lower saddle heights. Additionally, in the +7% condition, the knee and hip joints in the AL achieved the highest extension, deviating the most from those of the control. This extension (14° for the knee joint) was significantly beyond normal levels and may have hindered the participants’ ability to use the AL effectively. Therefore, the participants may have preferred using the UL, which was in an optimal position within the 25–35° range.

Additionally, in the fourth quarter on the AL side and at +7% height, the semitendinosus muscle (Figs 5B and 6B) activity in the AL was increased by over 300%. A moderate increase in semitendinosus activity under similar conditions has been reported before [10] and is compatible with the knee flexor patterns. However, the movement analysis showed that the +7% saddle height had the lowest maximum knee flexion (Fig 3). This peak semitendinosus activity might be related to the muscle performing at an alternate force-length setting [38]. Another possibility is the use of a clipless pedal attachment for the AL during the experiment, leading the participants to employ more muscle force during knee flexion at higher saddle heights to pull the pedal up at the end of the cycle. Higher hamstring activity during the last quarter of the cycle was documented when pulling clipless pedals [39]. In this context, it might be used to compensate for reduced muscle activity in the knee extensor muscles of the AL such as the vastus medialis, shifting peak power production from pushing down to pulling up during an upstroke.

#### Power delivery and symmetry

The effects described for the muscle activity and joint movement were directly reflected in the data collected by the instrumented pedals. At +7% height, the UL was responsible for 79% of the power production (Fig 7). This height also resulted in increased torque effectiveness (Fig 8A) and power smoothness (Fig 8B) in the UL, whereas both parameters were decreased in the AL. The increased power asymmetry, difference in pedaling technique, and higher extension of the AL affected the perceived exertion (Fig 9), with higher saddle heights being rated as the most strenuous. Therefore, it can be concluded that at increased saddle heights, the pedaling technique mainly employs the UL. The sound side becomes the main driver of the cycling movement, thereby increasing the asymmetry between the AL and UL sides.

### Effects of lowering the saddle height

Although lower saddle heights are associated with knee pain and other negative factors in competitive cycling [32], cyclists may choose lower the saddle for commuting and leisure activities. This choice provides a lower center of gravity, which enhances balance and facilitates starting and stopping, which is often necessary in urban environments [18]. Consequently, the control saddle height in this study targeted a maximum knee extension angle of 45°. Under the simulated prosthesis conditions, lower saddle heights brought the kinematic and kinetic parameters of the AL closer to those of the control condition.

#### Changes in pedaling technique

The -7% condition showed knee angles throughout the cycle that matched those in the control condition (Fig 3). The simulated prosthesis condition prevents ankle movement, which typically provides extra reach and force at the lowest pedal position. Therefore, the 0% saddle height shows increased extension, which decreases with lower saddle heights, closely matching the control at -7%. However, for hip angles (Fig 4), the -7% height deviated the most from the control at the beginning and end of the cycle. With the pedal in its highest position, the ankle cannot dorsiflex for clearance, resulting in a higher hip flexion. However, the reduced distance between the saddle and crank spindle at lower saddle heights diminished hip overextension. Consequently, at -3.5%, the hip angle matched the maximum hip extension of the control condition. This aligns with the findings of a previous study on joint movement in triathletes and cyclists at different saddle heights, in which the knee and hip joints showed increased flexion at lower saddle heights [16].

Both vastus medialis and semitendinosus muscles (Figs 5B, 5C, 6B and 6C) showed results closer to those in the control condition at lower saddle heights throughout the cycle for both UL and AL. Because these muscles contribute to knee motion as knee extensors and flexors, respectively, the observed muscle activity levels can be related to knee angles being closest to the control at -7% height. However, lower saddle heights resulted in activation levels below the control during the second quarter of the cycle in the gastrocnemius medialis muscle (Figs 5A and 6A) in the UL. The ankle joint has a limited range of dorsiflexion (10–20°), with most of its motion in plantarflexion (40–55°) [40]. With a lower saddle height, the ankle joint in the UL may enter a range where only dorsiflexion is required and plantarflexion becomes restricted [41]. Therefore, the ankle power production shifts to the knee and hip joints, making the UL resemble a prosthetic condition in which the ankle joint is absent [4, 42].

#### Power delivery and symmetry

Lower saddle heights resulted in knee and ankle movements and muscle activity in the proximal portion of the legs that closely matched the control conditions, suggesting better power symmetry. At -7% saddle height, the UL produced 58% of power (Fig 7), indicating near-symmetric power production. Similar findings in intact cycling [43] showed greater pedaling power but reduced force effectiveness at lower saddle heights. In this study, the torque effectiveness (Fig 8A) was increased in the AL at lower saddle heights, similar to the pedal smoothness results (Fig 8B). Despite better power symmetry at -7%, this was neither the least strenuous condition (Fig 9) nor the most comfortable (n = 1). The -3.5% condition had lower perceived exertion and was deemed most comfortable (n = 5). This may be related to ankle joint movement; increased dorsiflexion and restricted plantarflexion may cause discomfort. A previous study [21] found that comfort was decreased at lower saddle positions among recreational cyclists, with no significant difference between preferred and higher heights. Consistent with the results of the present study, saddle height did not significantly affect perceived exertion.

### Implications

Musculoskeletal injuries are prevalent among cyclists and may be exacerbated by lower saddle positions. However, this study suggests that for leisure-level cycling with unilateral prostheses, lower saddle heights are recommended. Since leisure-level cycling does not require the increased power output resultant of higher saddle positions, this population can focus on comfort and the enhanced symmetry in power application and reduced strain achieved by the lower saddle heights. Thus, it can be concluded that the optimal saddle height in this population should maintain the dynamically measured maximum knee extension of the affected side within the 37–45° range, corresponding to the -3.5% and -7% saddle height conditions used in this study.

Following these findings, healthcare professionals can prescribe lower saddle heights for patients willing to start cycling. Furthermore, when having a first contact with cycling practice, users with amputations can potentially be discouraged from cycling if using incorrected settings, thus making the activity uncomfortable or impossible to carry out. This may lead to the belief that they are unable to practice leisure cycling. This is also reflected in research results [44] which show that one of the main barriers for persons with amputations in taking up cycling is the inappropriate bicycle or prosthesis configuration. These findings can also inform the design of cycling equipment specifically directed at individuals with amputations. Beyond direct applications, the study of muscle activity changes while cycling can help inform the development of powered cycling prostheses which utilizing sEMG data for movement [45].

### Limitations

The use of actual lower-limb prostheses requires motor adaptation [31] and adjustment to limit muscle function after the procedure. However, these factors were not evaluated in this study. Additionally, the age range of the participants in this study was notably lower than that of the population with transtibial amputations that partake in cycling (62.0 ± 13.0 years) [44]; this was due to the introduction of the age-related factors (decline in muscle strength, endurance and balance, etc.), injury risk inferred by the task and, the possibility of exhaustion during the required physical activity. Future studies involving persons with lower-limb amputations will include middle-aged and older participants. A key element of the saddle height selection in the leisure cycling context is the actual daily use of bicycles and the maneuverability provided by the saddle height. Since this short study was conducted with a stationary bicycle, these adaptations and the development of a cycling technique could not be considered. Furthermore, the affected side was evaluated using the kinematic data. With the aim of collating data, a full evaluation of the unaffected side could provide further insights into the asymmetries.

## Conclusion

Lower saddle heights resulted in joint movement and muscle activity levels more closely resembling those in control conditions and improved power symmetry between the AL and UL. Thus, adopting lower saddle heights in leisure cycling is a feasible and straightforward modification of the cycling equipment for individuals with unilateral transtibial prostheses.

## Supporting information

S1 File Full results. (XLSM)

S1 Appendix Dunnett’s test results. (PDF)

## References

[1] Deans S, Burns D, McGarry A, Murray K, Mutrie N. Motivations and barriers to prosthesis users participation in physical activity, exercise and sport: A review of the literature. Prosthet Orthot Int. 2012;36: 260–9. doi: 10.1177/0309364612437905 22918902

[2] Ülger Ö, Yıldırım Şahan T, Çelik SE. A systematic literature review of physiotherapy and rehabilitation approaches to lower-limb amputation. Physiother Theory Pract. 2018;34: 821–834. doi: 10.1080/09593985.2018.1425938 29351504

[3] Poonsiri J, Dekker R, Dijkstra PU, Hijmans JM, Geertzen JHB. Bicycling participation in people with a lower limb amputation: a scoping review. BMC Musculoskeletal Disorders. 2018;19: 398. doi: 10.1186/s12891-018-2313-2 30424748 PMC6234608

[4] Childers WL, Kistenberg RS, Gregor RJ. The biomechanics of cycling with a transtibial amputation: Recommendations for prosthetic design and direction for future research. Prosthet Orthot Int. 2009;33: 256–271. doi: 10.1080/03093640903067234 19658015

[5] Johnston TE. Biomechanical Considerations for Cycling Interventions in Rehabilitation. Phys Ther. 2007;87: 1243–1252. doi: 10.2522/ptj.20060210 17636157

[6] Behrendt C-A, Sigvant B, Szeberin Z, Beiles B, Eldrup N, Thomson IA, et al. International Variations in Amputation Practice: A VASCUNET Report. Eur J Vasc Endovasc Surg. 2018;56: 391–399. doi: 10.1016/j.ejvs.2018.04.017 29859821

[7] Dillingham TR, Pezzin LE, MacKenzie EJ. Limb amputation and limb deficiency: epidemiology and recent trends in the United States. South Med J. 2002;95: 875–883. doi: 10.1097/00007611-200208000-00018 12190225

[8] Bussmann JB, Grootscholten EA, Stam HJ. Daily physical activity and heart rate response in people with a unilateral transtibial amputation for vascular disease. Arch Phys Med Rehabil. 2004;85: 240–244. doi: 10.1016/s0003-9993(03)00485-4 14966708

[9] Lower Limb Amputations: Epidemiology and Assessment | PM&R KnowledgeNow. 1 Mar 2017 [cited 28 Jun 2024]. Available: https://now.aapmr.org/lower-limb-amputations-epidemiology-and-assessment/

[10] Seratiuk Flores H, Yeoh WL, Loh PY, Morinaga K, Muraki S. Biomechanical Analysis of Recreational Cycling with Unilateral Transtibial Prostheses. Prosthesis. 2023;5: 733–751. doi: 10.3390/prosthesis5030052

[11] Pierson-Carey CD, Brown DA, Dairaghi CA. Changes in resultant pedal reaction forces due to ankle immobilization during pedaling. J Appl Biomech. 1997;13: 334–346. doi: 10.1123/jab.13.3.334

[12] Morgenroth DC, Gellhorn AC, Suri P. Osteoarthritis in the Disabled Population: A Mechanical Perspective. PM R. 2012;4: S20–S27. doi: 10.1016/j.pmrj.2012.01.003 22632698

[13] Devan H, Hendrick P, Ribeiro DC, A Hale L, Carman A. Asymmetrical movements of the lumbopelvic region: Is this a potential mechanism for low back pain in people with lower limb amputation? Med Hypotheses. 2014;82: 77–85. doi: 10.1016/j.mehy.2013.11.012 24296234

[14] Koutny D, Paloušek D, STOKLÁSEK P, Rosický J, Tepla L, Prochazkova M, et al. The biomechanics of cycling with transtibial prosthesis: a case study of a professional cyclist. International Journal of Medical, Health, Biomedical, Bioengineering and Pharmaceutical Engineering. 2013;7: 476–481.

[15] Robergs R, Peveler W, Bishop P, Smith J, Richardson M. Comparing methods for setting saddle height in trained cyclists. 2005;8.

[16] Bini RR, Hume PA, Kilding AE. Saddle height effects on pedal forces, joint mechanical work and kinematics of cyclists and triathletes. Eur J Sport Sci. 2014;14: 44–52. doi: 10.1080/17461391.2012.725105 24533494

[17] Bini R, Priego-Quesada J. Methods to determine saddle height in cycling and implications of changes in saddle height in performance and injury risk: A systematic review. J Sports Sci. 2022;40: 386–400. doi: 10.1080/02640414.2021.1994727 34706617

[18] Burke E. High-tech Cycling. Human Kinetics; 2003.

[19] van Melick N, Meddeler BM, Hoogeboom TJ, Nijhuis-van der Sanden MWG, van Cingel REH. How to determine leg dominance: The agreement between self-reported and observed performance in healthy adults. PLoS One. 2017;12: e0189876. doi: 10.1371/journal.pone.0189876 29287067 PMC5747428

[20] de Vey Mestdagh K. Personal perspective: in search of an optimum cycling posture. Appl Ergon. 1998;29: 325–334. 9703347

[21] Bini RR. Acute effects from changes in saddle height in perceived comfort during cycling. Int J Sports Sci Coach. 2020;15: 390–397. doi: 10.1177/1747954120918965

[22] Yeoh WL, Seratiuk Flores H. Motion Analysis 2D. CPS Lab (Saga University); 2024. Available: https://github.com/cps-lab-saga/motion-analysis-2d

[23] Lukežič A, Vojíř T, Čehovin L, Matas J, Kristan M. Discriminative Correlation Filter with Channel and Spatial Reliability. Int J Comput Vis. 2018;126: 671–688. doi: 10.1007/s11263-017-1061-3

[24] Hermens HJ, Freriks B, Disselhorst-Klug C, Rau G. Development of recommendations for SEMG sensors and sensor placement procedures. J Electromyogr Kinesiol. 2000;10: 361–374. doi: 10.1016/s1050-6411(00)00027-4 11018445

[25] Childers L, Hodson-Tole E, Gregor R. Activation Changes In The Gastrocnemius Muscle: Adaptation To A New Functional Role Following Amputation.: 1077. Medicine and Science in Sports and Exercise—MED SCI SPORT EXERCISE. 2009;41. doi: 10.1249/01.mss.0000354161.24516.91

[26] Childers WL, Kistenberg RS, Gregor RJ. Pedaling asymmetries in cyclists with unilateral transtibial amputation: effect of prosthetic foot stiffness. J Appl Biomech. 2011;27: 314–321. doi: 10.1123/jab.27.4.314 21896953

[27] Burden AM, Trew M, Baltzopoulos V. Normalisation of gait EMGs: a re-examination. J Electromyogr Kinesiol. 2003;13: 519–532. doi: 10.1016/s1050-6411(03)00082-8 14573367

[28] Bini RR, Hume PA. Assessment of bilateral asymmetry in cycling using a commercial instrumented crank system and instrumented pedals. Int J Sports Physiol Perform ce. 2014;9: 876–881. doi: 10.1123/ijspp.2013-0494 24509507

[29] Borg G. Perceived exertion as an indicator of somatic stress. Scand J Rehabil Med. 1970;2: 92–98. 5523831

[30] LeMond G, Gordis K. Greg LeMond’s Complete Book of Bicycling. Putnam; 1987.

[31] Childers WL, Prilutsky BI, Gregor RJ. Motor adaptation to prosthetic cycling in people with trans-tibial amputation. J Biomech. 2014;47: 2306–2313. doi: 10.1016/j.jbiomech.2014.04.037 24818794 PMC4076118

[32] Bini R, Hume PA, Croft JL. Effects of bicycle saddle height on knee injury risk and cycling performance. Sports Med. 2011;41: 463–476. doi: 10.2165/11588740-000000000-00000 21615188

[33] Priego Quesada JI, Pérez-Soriano P, Lucas-Cuevas AG, Salvador Palmer R, Cibrián Ortiz de Anda RM. Effect of bike-fit in the perception of comfort, fatigue and pain. J Sports Sci. 2017;35: 1459–1465. doi: 10.1080/02640414.2016.1215496 27490817

[34] Dyer B. Cycling with an amputation: A systematic review. Prosthet Orthot Int. 2016;40: 538–544. doi: 10.1177/0309364615610659 26527756

[35] Connick MJ, Li F-X. The impact of altered task mechanics on timing and duration of eccentric bi-articular muscle contractions during cycling. Journal of Electromyography and Kinesiology. 2013;23: 223–229. doi: 10.1016/j.jelekin.2012.08.012 23010605

[36] Verma R, Hansen EA, de Zee M, Madeleine P. Effect of seat positions on discomfort, muscle activation, pressure distribution and pedal force during cycling. J Electromyogr Kinesiol. 2016;27: 78–86. doi: 10.1016/j.jelekin.2016.02.003 26938676

[37] Ericson MO, Nisell R, Arborelius UP, Ekholm J. Muscular activity during ergometer cycling. Scand J Rehabil Med. 1985;17: 53–61. 4023660

[38] da Silva JCL, Tarassova O, Ekblom MM, Andersson E, Rönquist G, Arndt A. Quadriceps and hamstring muscle activity during cycling as measured with intramuscular electromyography. Eur J Appl Physiol. 2016;116: 1807–1817. doi: 10.1007/s00421-016-3428-5 27448605 PMC4983295

[39] Mornieux G, Stapelfeldt B, Gollhofer A, Belli A. Effects of pedal type and pull-up action during cycling. Int J Sports Med. 2008;29: 817–822. doi: 10.1055/s-2008-1038374 18418807

[40] Brockett CL, Chapman GJ. Biomechanics of the ankle. Orthop Trauma. 2016;30: 232–238. doi: 10.1016/j.mporth.2016.04.015 27594929 PMC4994968

[41] Moreno-Pérez V, Courel-Ibáñez J, Mateo-March M, López-Samanes Á, Del Coso J. Descriptive profile for lower-limb range of motion in professional road cyclists. J Sports Med Phys Fitness. 2021;61. doi: 10.23736/S0022-4707.20.11178-2 32880133

[42] Childers WL, Kogler GF. Symmetrical kinematics does not imply symmetrical kinetics in people with transtibial amputation using cycling model. J Rehabil Res Dev. 2014;51: 1243–1254. doi: 10.1682/JRRD.2013.11.0241 25629527

[43] Bini RR, Hume PA, Crofta JL. Effects of saddle height on pedal force effectiveness. Procedia Engineering. 2011;13: 51–55. doi: 10.1016/j.proeng.2011.05.050

[44] Poonsiri J, Dekker R, Dijkstra PU, Hijmans JM, Geertzen JHB. Cycling in people with a lower limb amputation. BMC Sports Sci Med Rehabil. 2021;13: 75. doi: 10.1186/s13102-021-00302-3 34246299 PMC8272388

[45] Mobarak R, Tigrini A, Verdini F, Al-Timemy AH, Fioretti S, Burattini L, et al. A Minimal and multi-source recording setup for ankle joint kinematics estimation during walking using only proximal information from lower limb. IEEE Trans Neural Syst Rehabil Eng. 2024;32: 812–821. doi: 10.1109/TNSRE.2024.3364976 38335075

