# Correction: Biomechanical effects of saddle height changes in leisure cycling with unilateral transtibial prostheses: A simulated study

**DOI:** 10.1371/journal.pone.0321575

**Published:** 2025-04-01

**Authors:** Heloisa Seratiuk Flores, Wen Liang Yeoh, Ping Yeap Loh, Kosuke Morinaga, Satoshi Muraki

The second author’s first and last name are incorrectly switched. The correct name is: Wen Liang Yeoh.

The third author’s name is spelled incorrectly. The correct name is: Ping Yeap Loh.

The correct citation is: Seratiuk Flores H, Yeoh WL, Loh PY, Morinaga K, Muraki S (2025) Biomechanical effects of saddle height changes in leisure cycling with unilateral transtibial prostheses: A simulated study. PLoS ONE 20(1): e0317121. https://doi.org/10.1371/journal.pone.0317121.

The ORCID iDs are missing for the second, fourth and fifth author. Please see the authors’ respective ORCID iDs here:

- Author Wen Liang Yeoh’s ORCID iD is: 0000-0003-1272-9010 (https://orcid.org/ 0000-0003-1272-9010).
- Author Kosuke Morinaga’s ORCID iD is: 0000-0002-9709-9902 (https://orcid.org/ 0000-0002-9709-9902).
- Author Satoshi Muraki’s ORCID iD is: 0000-0002-7912-9012 (https://orcid.org/ 0000-0002-7912-9012).
