# Supplementary material for: Biomechanical effects of saddle height changes in leisure cycling with unilateral transtibial prostheses: A simulated study
Source: PLoS One. 2025 Jan 7;20(1):e0317121. doi: 10.1371/journal.pone.0317121 (PMC11706476; doi:10.1371/journal.pone.0317121)
Supplement: S1 Appendix — (PDF) [file pone.0317121.s002.pdf]

Appendix 1. Significant Dunnett's test results.

Joint angles

Variable	Saddle Height	Mean Difference	Significance
**Knee 0°**	-3.5%	-5.14	.000
	0	-8.55	.000
	+3.5%	-12.32	.000
	+7%	-15.27	.000
**Knee 90°**	-3.5%	-4.64	.000
	0	-9.13	.000
	+3.5%	-13.71	.000
	+7%	-17.89	.000
**Knee 180°**	-3.5%	-10.18	.000
	0	-18.76	.000
	+3.5%	-26.46	.000
	+7%	-33.05	.000
**Knee 270°**	-3.5%	-5.92	.000
	0	-10.73	.000
	+3.5%	-15.54	.000
	+7%	-19.32	.000
**Hip 0°**	-7%	-10.54	.000
	-3.5%	-7.50	.000
	0	-5.08	.000
	+3.5%	-2.04	.004
**Hip 90°**	-7%	-9.89	.000
	-3.5%	-6.58	.000
	0	-3.60	.000
	+3.5%	-1.67	.004
	+7%	2.28	.000
**Hip 180°**	-7%	-5.37	.000
	0	4.46	.000
	+3.5%	9.02	.000
	+7%	13.34	.000

Variable	Saddle Height	Mean Difference	Significance
**Hip 270°**	-7%	-7.01	.000
	-3.5%	-4.53	.000
	0	-1.97	.001
	+3.5%	2.23	.000
	+7%	4.06	.000

Instrumented pedals

Variable	Saddle Height	Mean Difference	Significance
**L/R Balance**	-7%	9.77	.006
	-3.5%	14.33	.000
	0	20.62	.000
	+3.5%	20.88	.000
	+7%	30.90	.000
**Unaffected Leg Torque Effectiveness**	-3.5%	4.75	.047
	0	7.89	.000
	+3.5%	11.14	.000
	+7%	17.15	.000
**Affected Leg Torque Effectiveness**	-7%	-8.25	.004
	-3.5%	-10.48	.000
	0	-11.79	.000
	+3.5%	-11.63	.000
	+7%	-14.70	.000
**Unaffected Leg Pedal Smoothness**	0	1.83	.017
	+3.5%	2.80	.000
	+7%	3.98	.000
**Affected Leg Pedal Smoothness**	-7%	-2.09	.008
	-3.5%	-2.78	.000
	0	-2.84	.000
	+3.5%	-2.99	.000
	+7%	-3.60	.000

Subjective evaluation

Variable	Saddle Height	Mean Difference	Significance
**Borg Scale**	-7%	3.11	.000
	-3.5%	2.21	.001
	0	2.28	.001
	+3.5%	3.55	.000
	+7%	4.57	.000
